# Advances in Optical Biosensors for Pesticide Detection

**DOI:** 10.34133/research.1060

**Published:** 2026-01-09

**Authors:** Meini Zhao, Yijie Wang, Rui Jin, Hongxia Li, Xingguang Su, Xu Yan

**Affiliations:** ^1^ Department of Food Quality and Safety College of Food Science and Engineering, Jilin University, Changchun 130062, P. R. China.; ^2^ State Key Laboratory on Integrated Optoelectronics, Key Laboratory of Advanced Gas Sensors of Jilin Province, College of Electronic Science & Engineering, Jilin University, Changchun 130012, P. R. China.; ^3^ Department of Analytical Chemistry, College of Chemistry, Jilin University, Changchun 130012, P. R. China.

## Abstract

The growing imperative to ensure food safety, preserve ecological integrity, and mitigate public health risks associated with pesticide residues has driven a critical demand for highly sensitive optical sensors. In this regard, optical biosensors, including fluorescence (FL), colorimetry (CL), surface-enhanced Raman scattering (SERS), surface plasmon resonance (SPR), and chemiluminescence strategies, have been developed for pesticide detection. This review aims to provide a comprehensive summary of both fundamental knowledge and advancements in the field of optical biosensors for pesticide detection. The advantages of these biosensors are highlighted, such as excellent sensitivity, high specificity, and on-site application. Subsequently, a detailed overview of the sensing mechanism of optical biosensors based on different molecular recognition elements [e.g., enzymes, antibodies, aptamers, molecularly imprinted polymers (MIPs), and supramolecular host–guest complexes] is provided. Finally, perspectives are offered on the current challenges and future directions of pesticide biosensors. This review is expected to serve as a fundamental guide for researchers in the field of optical biosensors for pesticide detection and to provide insights and avenues to enhance the performance of existing sensing mechanisms in applications across diverse fields.

## Introduction

Pesticides play a crucial role in supporting the rapid development and high productivity of modern industrialized agriculture. By effectively controlling pests, weeds, fungi, and pathogens, they markedly increase crop yields and help maintain a stable food supply for a growing global population. Therefore, the use of pesticides in modern agriculture is an inevitable trend. However, the extensive application of pesticides has led to widespread residue accumulation in soil, water sources, and food products, raising serious concerns regarding food safety and ecological health. Numerous studies have confirmed that pesticide can bioaccumulate through the food chain, leading to adverse health effects such as neurotoxicity, endocrine disruption, and increased cancer risks [1,2]. Annual global reports attribute tens of thousands of foodborne illnesses to pesticide exposure [3,4]. These pressing issues highlight the urgent need for developing rapid, highly sensitive, and field-deployable pesticide detection technologies to ensure environmental safety and ecological health.

Conventional methods for pesticide detection, such as high-performance liquid chromatography (HPLC) and gas chromatography–mass spectrometry (GC-MS), are recognized for their high accuracy, reliability, and sensitivity. These techniques remain the gold standard for regulatory compliance and residue verification. Nonetheless, they involve sophisticated instrumentation, require extensive sample preparation, and often entail prolonged analysis time ranging from several hours to days, which hinders their use for on-site monitoring. Additionally, these methods depend heavily on specialized operational skills and well-equipped laboratory settings, limiting their applicability in resource-limited environments or large-scale decentralized monitoring programs [5–7]. Such drawbacks emphasize the demand for alternative analytical platforms that combine high performance with practicality for field applications.

In response to these challenges, optical sensing technologies have emerged as promising tools for pesticide detection, offering notable advantages such as high sensitivity, operational simplicity, and on-site application. These systems function by transducing molecular recognition events into measurable optical signals, which can encompass a range of spectral changes, such as variations in absorption, reflectance, or fluorescence (FL) emission. For instance, FL-based sensors exploit the properties of probes like quantum dots (QDs), carbon dots (CDs), gold nanoclusters (AuNCs), and organic dyes to detect pesticides at ultralow concentrations through FL quenching or enhancement [8,9]. Colorimetry (CL) detection operates by transducing analyte concentration into measurable changes in absorbance intensity via catalytic chromogenic reactions, thereby facilitating rapid, cost-effective, and stable detection without reliance on sophisticated instrumentation [10,11]. Furthermore, surface-enhanced Raman scattering (SERS) leverages plasmonic nanostructures to amplify Raman signals dramatically, allowing precise identification of pesticide molecules via their unique vibrational fingerprints, even in complex matrices [12]. The technique’s minimal matrix interference and compatibility with portable spectrometers facilitate multiplexed detection in real-world agricultural and environmental samples.

**Fig. 1.**
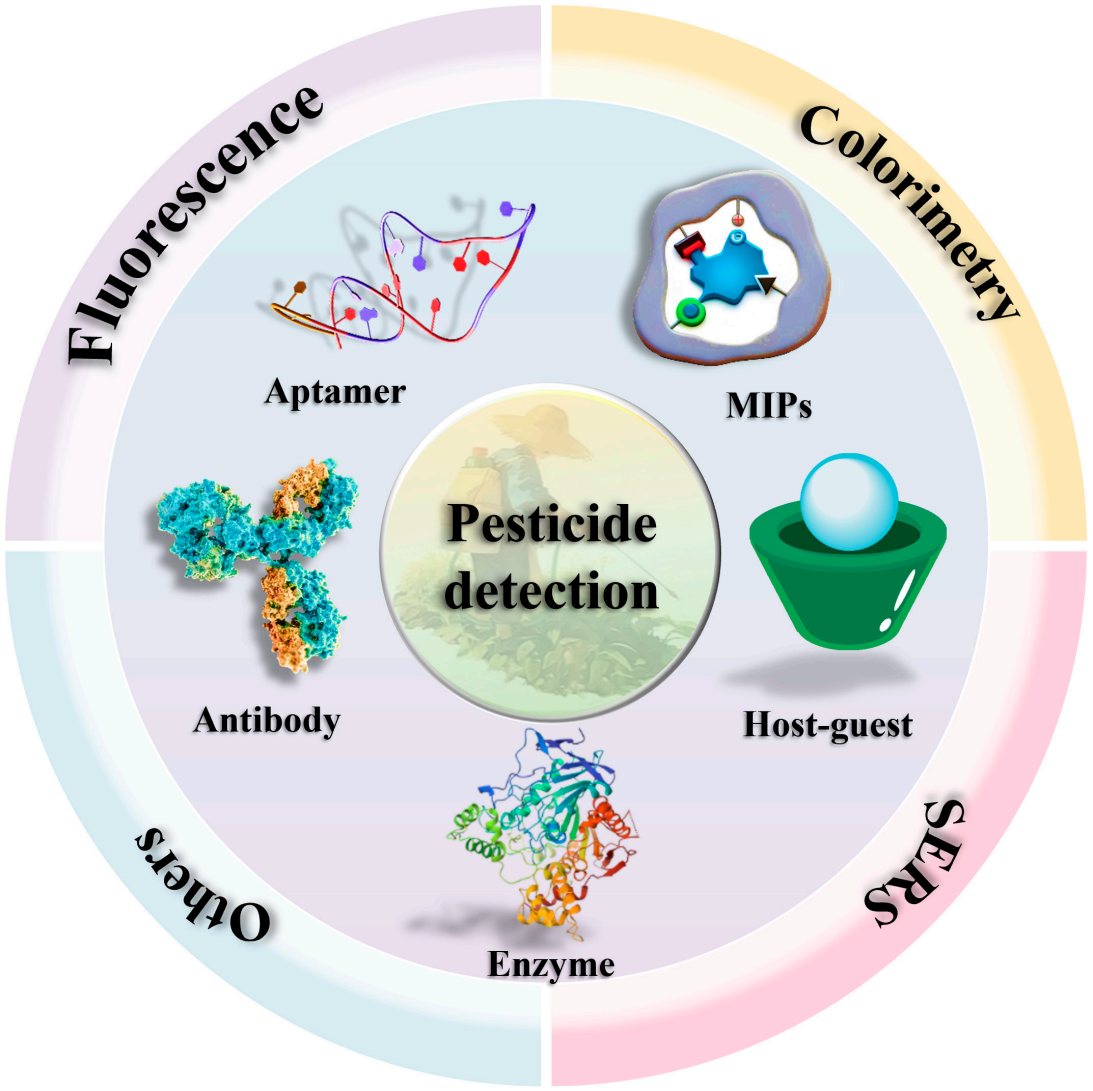
Schematic of different optical sensors for pesticide detection based on recognition elements.

Despite significant progress, the practical implementation of optical sensing platforms still faces several challenges. These include ensuring the stability and reproducibility of functional nanomaterials, integrating sensing elements into robust and user-friendly devices, and improving selectivity in highly complex sample matrices [13,14]. This review comprehensively summarizes recent advances in optical sensing strategies, including FL, CL, and SERS for pesticide detection (Fig. 1). It also provides a detailed analysis of various biorecognition elements [e.g., enzymes, antibodies, aptamers, molecularly imprinted polymers (MIPs), and host–guest systems], which are instrumental in enhancing analytical performance, enabling detection limits as low as fg/ml, as well as improving specificity and matrix tolerance. We critically discuss the role of functional nanomaterials such as QDs, metal–organic frameworks (MOFs), and nanozymes in facilitating ultrasensitive and portable detection. Finally, we outline emerging trends and prospects for the development of intelligent, connected sensing systems for real-time pesticide monitoring and environmental surveillance.

## Typical Optical Sensor Strategies

### FL sensing strategies

FL sensors represent powerful tools for pesticide detection due to their high sensitivity, exceptional specificity, and rapid response, enabling precise quantification even at trace concentrations. FL sensors often use enzymes, antibodies, aptamers, MIPs, and host–guest complexes as recognition elements. These elements are often functionalized with various FL nanomaterials, such as QDs, CDs, and AuNCs, to improve detection capabilities. When these functionalized composites interact with target pesticides, they exhibit significant changes in FL emission, thereby enabling effective pesticide detection at trace levels. This section will focus on the latest progress and current challenges of FL sensors based on these 5 types of recognition elements in pesticide detection.

#### Enzyme-mediated methods

**Fig. 2.**
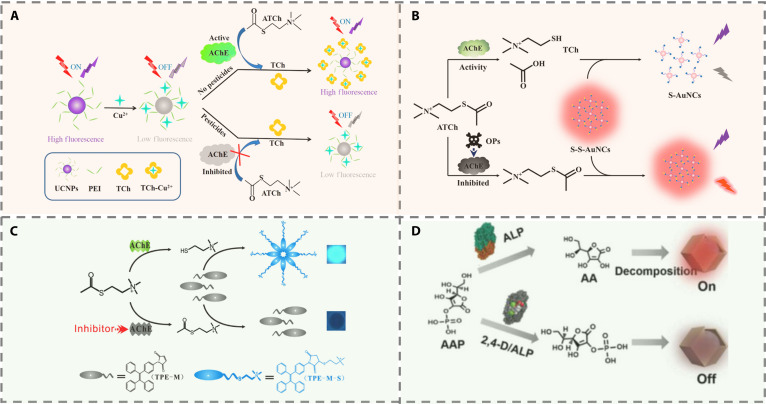
(A) Schematic description of the acetylcholinesterase-modulated UCNPs-Cu^2+^ FL biosensor for organophosphorus pesticides [16]. Copyright © 2019, American Chemical Society. (B) Diagrammatic sketch of the sensing mechanism of S-S-AuNCs for OPs [17]. Copyright © 2021, American Chemical Society. (C) Schematic illustration of the detection principle of the as-proposed fluorescent assay for AChE activity and OPs [18]. Copyright © 2016 Elsevier B.V. All rights reserved. (D) Schematic depicting the 2,4-D detection principle [19]. Copyright © 2024 Wiley-VCH GmbH.

Enzymes offer significant advantages such as high substrate specificity, exceptional catalytic efficiency, and excellent biocompatibility. The integration of enzymes with FL nanomaterials preserves the molecular recognition capability of enzymes while incorporating the optical properties of nanomaterials, thereby substantially improving both the specificity and sensitivity of analyte detection. Several enzymes are widely used in pesticide detection (Table 1). These include acetylcholinesterase (AChE), alkaline phosphatase (ALP), organophosphorus hydrolase (OPH), urease, horseradish peroxidase (HRP), glucose oxidase (GOD), and Trp. Among them, AChE-based detection mechanisms are the most extensively established [15]. ATCh is hydrolyzed by AChE to produce thiocholine (TCh), which acts as a signaling mediator in the FL sensor. Due to its robust coordination capacity and reducing ability, TCh interacts with FL quenchers or indicators in the system, mitigating the quenching effect on FL probes and leading to FL recovery. For example, upconversion nanoparticles (UCNPs) possess FL quenching in the presence of Cu^2+^, whereas TCh can chelate Cu^2+^ ions, resulting in the FL restoration of UCNPs. The inhibition of AChE activity by pesticides prevents TCh generation, resulting in the reduction of FL recovery (Fig. 2A). This mechanism enables the detection of diazinon with a detection limit as low as 0.05 ng/ml [16]. Similarly, TCh can break disulfide bonds to trigger the disintegration of disulfide-functionalized AuNCs (S-S-AuNCs), leading to an FL reduction of the system. Meanwhile, acetic acid, another hydrolysis product of ATCh, further promotes FL attenuation of S-S-AuNCs (Fig. 2B). This approach allows the detection of organophosphorus pesticides (OPs) without external quenchers, thereby reducing detection costs [17]. A paper-based fluorescent sensor utilizes the enzyme AChE to hydrolyze ATCh, producing TCh (Fig. 2C). The thiol group of TCh then opens the maleimide ring of the AIEgen TPE-M, triggering strong FL. OPs inhibit AChE, reducing TCh production and the FL signal. This enables highly sensitive visual detection [18].

**Table 1.** Comparison of enzymes used in FL detection of pesticides and their characteristics

Enzyme	Target pesticide type	Characteristics	Reference
OPH	Organophosphates	Specifically degrades organophosphates, high detoxification efficiency	[15]
GOD	Organophosphates Carbamates	Specifically oxidizes β-d-glucose to produce gluconic acid	[15]
Tyr	Phenolic pesticides	Specifically catalyzes the oxidation of phenolic substrates to form quinone products	[15]
AChE	Organophosphates Carbamates	High sensitivity, wide application, easy to inactivate, poor selectivity	[16,17]
Urease	Heavy metals/partial pesticides	Commonly used for multi-analyte detection or catalyzing the formation of ammonia and carbon dioxide from urea, pH-sensitive	[18]
ALP	Phosphate monoesters	Optimal pH is alkaline, dependent on metal ions	[19]
HRP	Phenolic, aromatic amine pesticides	Rapidly catalyze the oxidation of substrates by peroxides, featuring a high reaction rate and excellent sensitivity	[63]
BChE	Organophosphates Carbamates	Wider substrate specificity than AChE, higher stability, poor selectivity	[67]

Recent advances in plant-wearable sensors have facilitated substantial progress in in situ pesticide monitoring, overcoming the constraints of field detection and enabling noninvasive, real-time surveillance. A notable example is an enzyme-mediated FL hydrogel disc. It incorporates UCNPs functionalized with a zeolitic imidazolate framework and polydopamine (UCNPs@ZIF@PDA) into a double-network hydrogel, enabling tight adhesion to plant surfaces. By exploiting the enzyme-mimetic catalytic activity of ZIF and the enzyme-responsive behavior of PDA, this system attains highly sensitive pesticide detection. The double-network hydrogel exhibits high transparency, mechanical flexibility, and strong adhesion, allowing it to conform closely to plant surfaces without disrupting stomatal function. The presence of the pesticide 2,4-dichlorophenoxyacetic acid (2,4-D) reduces the inhibition of ALP activity, which in turn reduces ascorbic acid (AA) production (Fig. 2D). This halts the decomposition of PDA and alters the FL signal of UCNPs, achieving a detection limit of 20 ng/ml. More intriguingly, the integration of smartphones and ImageJ software enables the conversion of FL signals into color Euclidean distance (ED) values for quantitative analysis, successfully tracking pesticide degradation dynamics on tomato plants [19]. Inspired by this strategy, our group used the spatial confinement architecture of ZIF-8 to trigger the aggregation-induced emission (AIE) effect of AuNCs, resulting in intrinsic signal enhancement and establishing a foundation for ultra-sensitive detection. Upon the decomposition of ZIF-8 by TCh, the AIE effect of AuNCs is broken, leading to FL changes that can be monitored via a smartphone. The projected signals (FL color) can be split into 3 primary color codes (RGB: red, green, blue) by the image-processing algorithm. This approach employs a B×R signal processing method, which capitalizes on the synergistic variation between the blue and red channel signals. The multiplicative operation markedly amplifies minor FL differences, thereby boosting the detection sensitivity to 0.2 ng/ml. The innovative B×R algorithm employs synergistic variations in blue and red dual-channel signals. Using multiplicative operations rather than simple distance mapping, this method significantly amplifies subtle FL differences, greatly improving detection sensitivity to 0.2 ng/ml. This platform allows the detection of trace-level pesticide residues, offering considerable practical value for early warning systems and meeting increasingly stringent food safety requirements [20].

Dual-emission ratiometric FL sensors utilize a target-responsive signal and a reference signal, quantifying analytes via intensity ratios to minimize environmental interference and enhance detection accuracy. By incorporating enzymes, this approach capitalizes on their high specificity for precise recognition, while the ratiometric signal ensures robust quantification. Offering high sensitivity, tunability, and ease of operation, this strategy overcomes limitations of conventional methods to detect trace analytes in complex samples, making it valuable in environmental, biomedical, and food safety monitoring [21]. For example, one ratiometric sensor capitalizes on the opposing FL responses of MnO_2_ nanosheets (MnO_2_ NSs) toward the scopoletin fluorophore and Amplex Red. While MnO_2_ NS quenches scopoletin FL, it simultaneously oxidizes Amplex Red to yield highly FL resorufin. The decomposition of MnO_2_ NS by TCh leads to the recovery of the scopoletin signal at 465 nm and a decrease in the resorufin emission at 585 nm, allowing ultrasensitive detection of OP pesticides with a detection limit as low as 1.6 pg/ml through the ratio F585/F465 [22]. Building on this concept, MnO₂ NS can also be incorporated into flexible hydrogels to create portable ratiometric detection kits suitable for on-site pesticide monitoring in agricultural settings, effectively reducing matrix interference. In one such system, an Au nanoclusters@manganese dioxide (AuNCs@MnO_2_) composite hydrogel, MnO_2_ again acts as a quencher and oxidant, inducing changes in the FL ratio between red-emitting AuNCs and yellow FL 2,3-diaminophenazine. When coupled with smartphone-based analysis, this platform enables dynamic on-site detection of OPs with a detection limit of 5.0 ng/ml [23].

Enzyme-based FL sensors leverage the inherent high specificity and catalytic efficiency of enzymes, combined with the high sensitivity of FL detection, to achieve the monitoring of trace pesticides. These sensors provide notable advantages, including signal amplification capability that enables ultra-low detection limits, excellent biocompatibility suitable for on-site detection, and versatile signal output modes compatible with smartphone-based quantification. Nevertheless, the long-term practical use of enzymes faces several challenges. These include their innate sensitivity to environmental factors like temperature and pH, along with their limited operational stability and reproducibility. Additionally, matrix interference in complex samples can compromise accuracy. Despite these limitations, ongoing advances in nanomaterial engineering and signal processing algorithms continue to support the promising potential of sensors in environmental surveillance and food safety monitoring.

#### Antibody-assisted methods

**Fig. 3.**
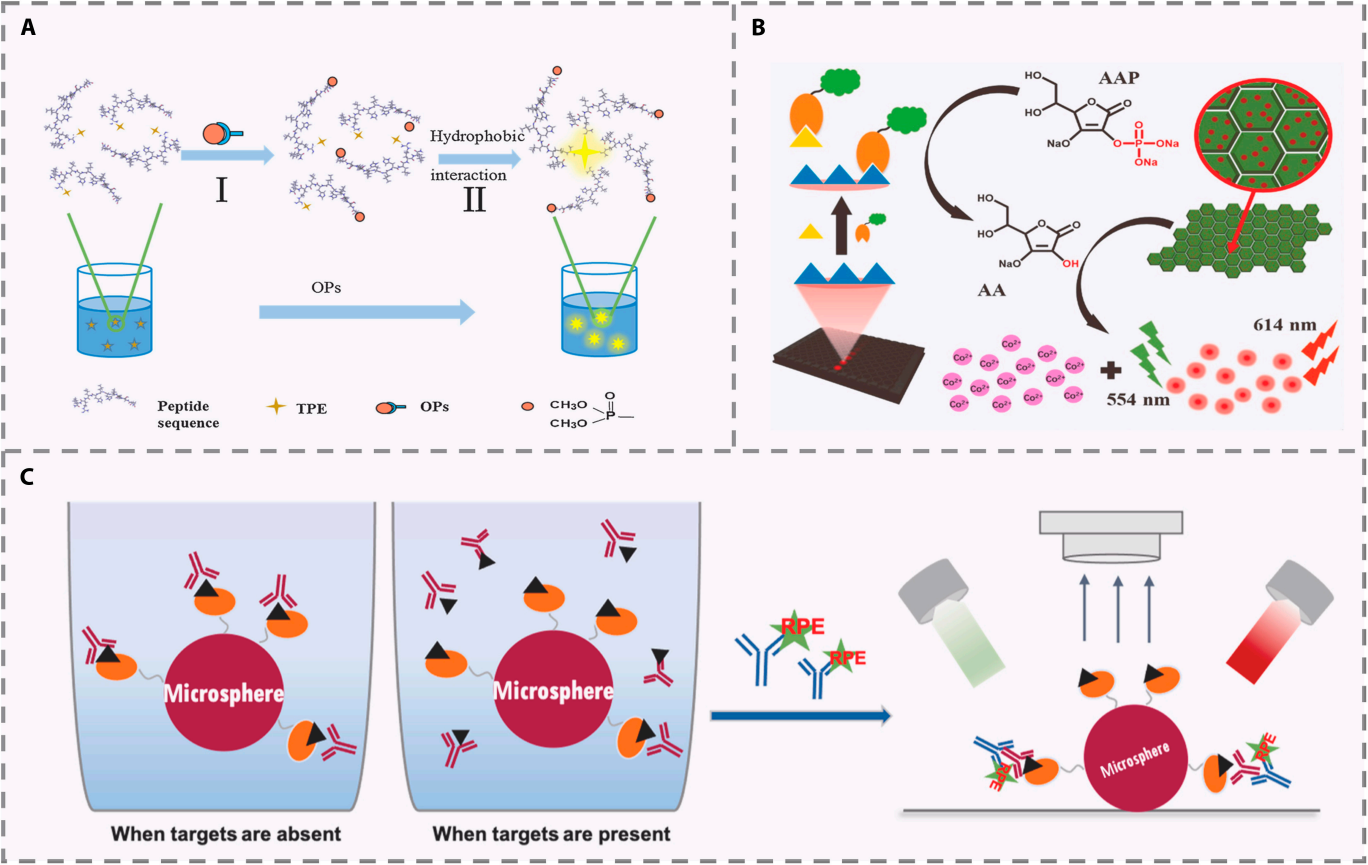
(A) Schematic diagram of TPE-peptide working principle [25]. Copyright © 2020 Elsevier B.V. All rights reserved. (B) Principle of the FL ELISA for detection of fenitrothion [26]. Copyright © 2022 Elsevier B.V. All rights reserved. (C) Schematic diagram of ic-FMIA principle [28]. Copyright © 2021 Elsevier B.V. All rights reserved.

Unlike enzymes that act through catalytic reactions, antibodies recognize analytes with high fidelity via a “key-lock” binding mechanism, enabling highly specific capture of pesticides [24]. The integration of this recognition principle with FL sensing technologies offers considerable potential for ultrasensitive and quantitative analysis, particularly in the context of on-site detection. Research in antibody-based FL sensors has led to the development of various high-performance strategies that combine the specific binding of antibodies or their mimetics with the sensitivity of FL reporting systems. For instance, one approach utilized an antibody-functionalized peptide probe engineered with AIE characteristics. Upon interaction with OPs, the probe undergoes molecular aggregation, which enhances its FL and enables selective detection of the target (Fig. 3A) [25]. Another study employed phage display-derived peptide mimics of antibodies fused to FL proteins, creating stable recombinant tracers that compete with the analyte for binding sites. Subsequent enzymatic amplification using ALP and CoOOH nanosheets induced FL recovery of CDs, enhancing sensitivity and reproducibility (Fig. 3B) [26].

With growing practical requirements, single-analyte detection is often insufficient for multi-residue screening in complex samples. Increasing efforts focus on achieving multiplexed detection with high sensitivity through integrated signal amplification platforms. One example is a competitive immunoassay utilizing catalytic hairpin assembly (CHA) combined with gold nanoparticle (AuNP)-labeled antibodies and DNA–fluorophore conjugates, which achieved detection limits as low as 5.7 to 12 μg/ml for several OPs [27]. For on-site applications, a dual-signal immunochromatographic strip was developed using recombinant FL peptides and AuNPs, yielding both CL and FL readouts (Fig. 3C). This design improves reliability and sensitivity through an inner filter effect-mediated quenching and competitive binding mechanism, making it suitable for rapid field testing [28].

Despite different designs, antibodies or their mimics are essential for specific target recognition and signal conversion in these sensors. Advances in antibody engineering and nanomaterials have improved their performance in complex environments. However, challenges remain regarding stability under varying conditions and reproducibility across tests. Specifically, parameters such as antibody stability, potential cross-reactivity, signal interference, and limited dynamic range need to be addressed. The lack of standardized protocols also hinders consistent application. Solving these issues is crucial for the practical use of antibody-based FL sensors.

#### Aptamer-based methods

**Fig. 4.**
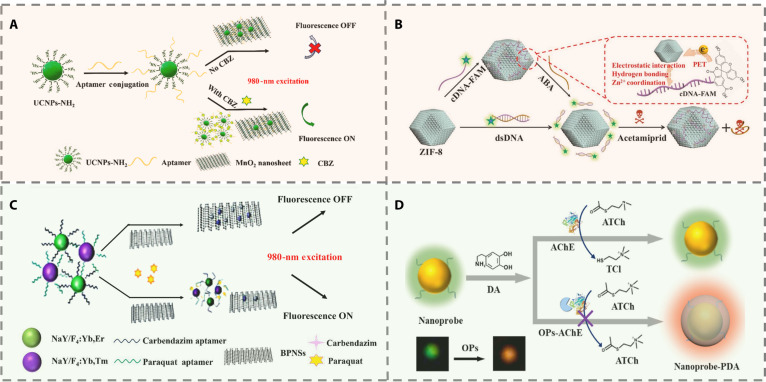
(A) Schematic description of FL nanosensor for carbendazim detection [31]. Copyright © 2021 Elsevier B.V. All rights reserved. (B) Principle of the FL ELISA for detection of fenitrothion [32]. Copyright © 2022 Elsevier B.V. All rights reserved. (C) Schematic diagram of the multiplexed FRET between UCNPs-Aptamer and black phosphorus nanosheet (BPNS) sensing platform for simultaneous detection of paraquat and carbendazim [34]. Copyright © 2021 Elsevier B.V. All rights reserved. (D) Schematic illustration of the FL sensor for OP assay [35]. Copyright © 2022 Elsevier B.V. All rights reserved.

Aptamers are single-stranded DNA or RNA molecules selected by systematic evolution of ligands by exponential enrichment (SELEX) [29]. They exhibit high specificity and affinity akin to antibodies, alongside advantages like facile synthesis, excellent stability, and ease of labeling [30]. These properties make them ideal for sensitive and cost-efficient detection of pesticides in food and environmental samples. The evolution of aptamer-based FL sensors reflects a clear trajectory from single-function assays toward integrated, multifunctional, and practical sensing platforms. In the early stages of development, research efforts were primarily directed at enhancing sensor performance through the incorporation of new nanomaterials. For example, an FL resonance energy transfer (FRET) system constructed from UCNPs and MnO_2_ NSs exploited aptamer conformational changes upon target binding to restore FL, enabling highly selective detection of carbendazim (Fig. 4A). This approach effectively mitigated background interference associated with conventional organic dyes and offered a new strategy for accurate pesticide residue analysis in complex matrices [31]. As research advanced, greater emphasis was placed on elucidating the molecular interaction mechanisms underlying signal transduction. For example, the MOF can adsorb DNA through multiple modes. These include electrostatic attraction, hydrogen bonding, and metal coordination. This property was used to build an FL sensor with a dual-signal conversion process (Fig. 4B). Such mechanism-driven designs not only improve detection reliability but also provide a theoretical foundation for precise signal modulation in complex systems [32].

With growing demands for practical applications, sensor designs have progressively evolved toward platform-based and generalized architectures to enable multiplex detection. Notable advances include the integration of magnetic separation technology with universal aptamer sequences for simultaneous detection of 3 OPs, significantly enhancing analytical efficiency [33]. Similarly, a dual-channel detection platform employing dual-color UCNPs and black phosphorus nanosheets to form a FRET pair allowed crosstalk-free analysis of paraquat (PQ) and carbendazim, representing a major step forward in multi-pesticide detection (Fig. 4C) [34]. More recently, aptamer-based FL sensors have exhibited trends toward multimodal sensing and functional integration. A prominent example is an FL-plasmonic resonance dual-mode platform utilizing AuNPs, which combines enzyme inhibition principles with aptamer-specific recognition (Fig. 4D). This system achieves highly sensitive detection of diverse pesticides and supports naked-eye visual interpretation, illustrating a diversification in detection methodologies and underscoring a shift from laboratory-based analysis toward field-deployable diagnostic devices [35].

In summary, aptamer-based FL sensor technology has evolved from single-analyte to multiplexed detection, and from offline to real-time monitoring. These improvements have enhanced food safety surveillance and show potential for use in more complex scenarios. However, challenges remain, such as the high cost of aptamer development, matrix effects, and limited portability. For practical use, stability under different conditions and reproducibility across tests must be improved. Future work should focus on creating scalable aptamer libraries, engineering more robust probes, and developing miniaturized devices for reliable field applications.

#### MIP-based methods

MIPs are a synthetic strategy for creating polymer scaffolds with tailored binding sites that complement the template molecules in size, shape, and functionality. They exhibit high chemical and thermal stability as well as strong resistance to harsh conditions. By employing template-directed polymerization of functional monomers, this technique allows the creation of polymer networks featuring spatially complementary cavities and tailored binding sites that demonstrate high specificity toward the template molecules. This selective recognition effectively minimizes interference from nontarget substances. MIP-based FL sensors combine the robust chemical stability and customizable molecular affinity of MIPs with the efficient signal transduction offered by optical reporters, enabling highly sensitive and selective detection of pesticides in complex food and environmental matrices [36].

Much of the research on MIP-based sensors aims to improve analytical performance through the design of the functional nanomaterials. For example, perovskite QDs (CsPbBr_3_) were incorporated into a MIP system. This achieved 2 goals: It enabled selective recognition of the target analyte and protected the QDs from solvent-induced quenching. As a result, the system achieved highly sensitive detection of phoxim. A key aspect of this approach was the use of a new silane monomer along with a controlled hydrolysis coating method, which enhanced both the density of recognition sites and the overall stability of the material [37]. In pursuit of accuracy and interference resistance of the platform, ratiometric FL sensing strategies have been developed. An example is a dual-emission system utilizing CDs, where blue-emitting imprinted CDs served as the responsive signal and red-emitting CDs acted as an internal reference. The binding of thiamethoxam increased blue FL, while the red signal remained constant, allowing quantitation based on the ratio of the 2 signals [38].

To address the growing need for field-deployable screening tools, MIP sensor designs have evolved from solution-based assays toward solid-supported and visual platforms. One notable example involved combining ultraviolet (UV)-induced FL fingerprinting with molecularly imprinted paper-based devices (MIP-PADs). A MIP layer fabricated on filter paper allowed UV-triggered emission of characteristic FL from target pesticides, thereby achieving on-site stable detection of multiple pesticides in apples. This approach minimizes sample pretreatment and is well-suited for in situ surface analysis of agricultural products [39]. As the detection of trace contaminants in complex samples advances, current research emphasizes not only high selectivity but also material sustainability and analytical throughput. For instance, an FL sensor with FeSe QDs in a MIP enables sensitive, selective, and rapid detection of cyfluthrin [40]. By integrating MIPs with dual-channel probes, simultaneous multi-pesticide detection is achieved, offering an efficient, high-throughput alternative to traditional enzyme-linked immunosorbent assay (ELISA) methods [41].

Aptamer-based FL sensing has evolved from single toward multiplexed detection, amplified signaling, and real-time monitoring, greatly improving food safety surveillance. However, challenges such as high aptamer development costs, sample matrix interference, and poor portability persist. Future efforts should prioritize scalable aptamer libraries, engineered probe stability, and miniaturized integrated devices to enable field-ready, robust detection platforms.

#### Host–guest interaction-based methods

**Fig. 5.**
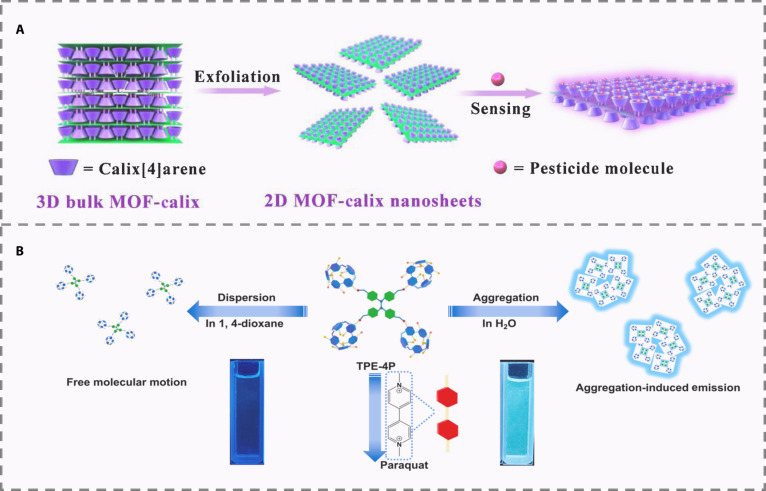
(A) Schematic illustration for the fabrication of 2D MOF-Calix nanosheets and sensitive detection of pesticide through host–guest chemistry [42]. Copyright © 2020 Elsevier B.V. All rights reserved. (B) Schematic illustration of paraquat sensing process [44]. Copyright © 2022 Elsevier B.V. All rights reserved.

Supramolecular materials achieve host–guest recognition via noncovalent interactions, modulating FL through conformational or energy transfer changes. Their specificity is tunable by designing cavity size, charge, and functional groups. Coupled with fluorophores, they form highly sensitive and rapid response sensors with promising applications in biomedical, environmental, and food safety fields. For example, a 2-dimensional layered MOFs functionalized with calix[4]arene (MOF-Calix) was developed to recognize glyphosate (Fig. 5A). Structural optimization and efficient exfoliation achieved a 32.3% yield and a rapid, selective FL enhancement response. This method relied on host–guest complexation-induced rigidification, enabling a detection limit of 2.25 μM that approaches drinking water standards, despite the system’s limited applicability [42]. Furthermore, cucurbit[7]uril (CB[7]) and an adamantane-functionalized rhodamine B probe were integrated to construct a reversible sensor for the detection of PQ. Through competitive supramolecular binding, this system achieved a cyclic “on–off” FL response with a detection limit as low as 10 nM. It was successfully applied in live-cell imaging and real agricultural sample analysis, demonstrating high selectivity, reversibility, and adaptability across multiple scenarios, thereby advancing the mechanism from static to dynamic recognition [43]. To overcome the aggregation-caused quenching (ACQ) effect of the fluorophore in MIP, another study integrated AIE characteristics with host–guest recognition, thereby realizing highly sensitive and stable detection of PQ through the synergistic effect of AIE and supramolecular complexation. A pillar[5]arene-modified tetraphenylethylene (TPE-4P) sensor was designed for PQ detection, exploiting AIE to avoid ACQ while using host–guest complexation to restrict molecular rotation and amplify emission (Fig. 5B). This system extended from solution detection to test strip-based semiquantification and in vivo imaging in zebrafish, illustrating enhanced application versatility and mechanistic sophistication [44].

Beyond broadening application scenarios, recent efforts have focused on functional integration and overcoming challenges associated with complex sample matrices. One example is a BODIPY-calixarene dual-mode sensor for sequential detection of Cu^2+^ and triazole pesticides. This platform utilized Cu-catalyzed azide-alkyne cycloaddition (CuAAC) click chemistry to graft BODIPY onto tetrazole-calixarene, forming a multi-armed recognition structure. Initial Cu^2+^ coordination with triazole groups induced chelation-enhanced quenching (CHEQ), while subsequent introduction of triazole pesticides displaced Cu^2+^ due to stronger affinity, restoring the FL of the system. This reversible dual-mode sensing strategy integrates CL and FL responses to achieve micromolar-level detection with high anti-interference capacity, providing a versatile platform for monitoring multiple pollutants in complex environments. This reversible dual-mode sensing strategy combines CL and FL responses. It achieved micromolar-level detection with high anti-interference capacity, thereby offering a versatile platform for monitoring multiple pollutants in complex environments [45].

Sensors based on supramolecular recognition benefit from straightforward rational design of host structures, avoiding complex biological screening or template removal steps. They are cost-effective and durable and respond within seconds to minutes. Easy integration with functional materials enhances their utility, while high sensitivity and biocompatibility support environmental and biological use. However, challenges remain, such as complex synthesis of host molecules, limited selectivity, and interference in mixed samples. For real-world use, stability under different conditions and reproducibility across repeated tests must be improved. Future efforts should focus on simplifying synthesis, reducing interference, and enhancing detection limits to achieve reliable performance.

#### Others

The development of material systems for FL sensors has evolved toward a coordinated multi-platform strategy. In addition to conventional recognition units (enzymes, antibodies, aptamers, and host–guest chemistry), MOFs and covalent organic frameworks (COFs) have also emerged as powerful candidates owing to their unique structural and functional advantages. MOFs offer highly designable porous architectures, ultrahigh specific surface areas, and excellent chemical stability, making them ideal as supporting matrices and recognition frameworks. For instance, rhodamine B is encapsulated within a cadmium-based MOFs to form the hybrid material, which exhibits dual emission of blue FL at 370 nm from the MOF scaffold and stable red FL from the encapsulated rhodamine B. Upon exposure to nitroaromatic pesticides such as acetamiprid, the blue FL of MOFs is selectively quenched. By monitoring the ratio of FL intensities (*I*₃₇₀/*I*₅₉₀), highly selective detection is achieved with a limit as low as 0.48 nM. The sensor retains its performance over 5 consecutive cycles, demonstrating significant reusability [46]. COFs, built from strong covalent bonds, exhibit high stability and tunable composition in comparison to other frameworks. A trifluoromethyl-functionalized COF-(CF_3_)_2_, synthesized via large-scale production, exhibits strong FL resulting from extended conjugation through cycloaddition. Simultaneously, it selectively adsorbs fluorinated pesticides such as trifluralin via fluorine–fluorine interactions. The resulting FL quenching follows Stern–Volmer kinetics, with detection reaching micromolar levels. This design integrates sample enrichment and detection into a single platform, enabling synergistic functionality [47].

These FL sensors improved sensitivity by using various biometric elements. However, key challenges remain. Photobleaching affects its long-term stability. Autofluorescence and quenching in complex samples reduce reliability. Some setups require expensive and complex instruments. Furthermore, spectral overlap limits its multiplex detection capability.

### CL sensing strategies

CL sensors operate by transducing the presence of target analytes into visually discernible color changes through a sequential process involving molecular recognition, signal development, and output interpretation [48]. These systems offer notable advantages, including operational simplicity, low cost, rapid response, and straightforward visual interpretation, making them particularly valuable for on-site screening applications [49,50]. A central challenge in the development of such platforms lies in the effective conversion of molecular binding events into clear and reliable CL responses [51,52]. Although various biorecognition elements can be employed, enzymes, antibodies, and aptamers constitute the most widely used classes for the construction of CL sensors. This section reviews recent advances and ongoing challenges related to CL pesticide detection platforms based on these 3 types of molecular receptors.

#### Enzyme-mediated methods

**Fig. 6.**
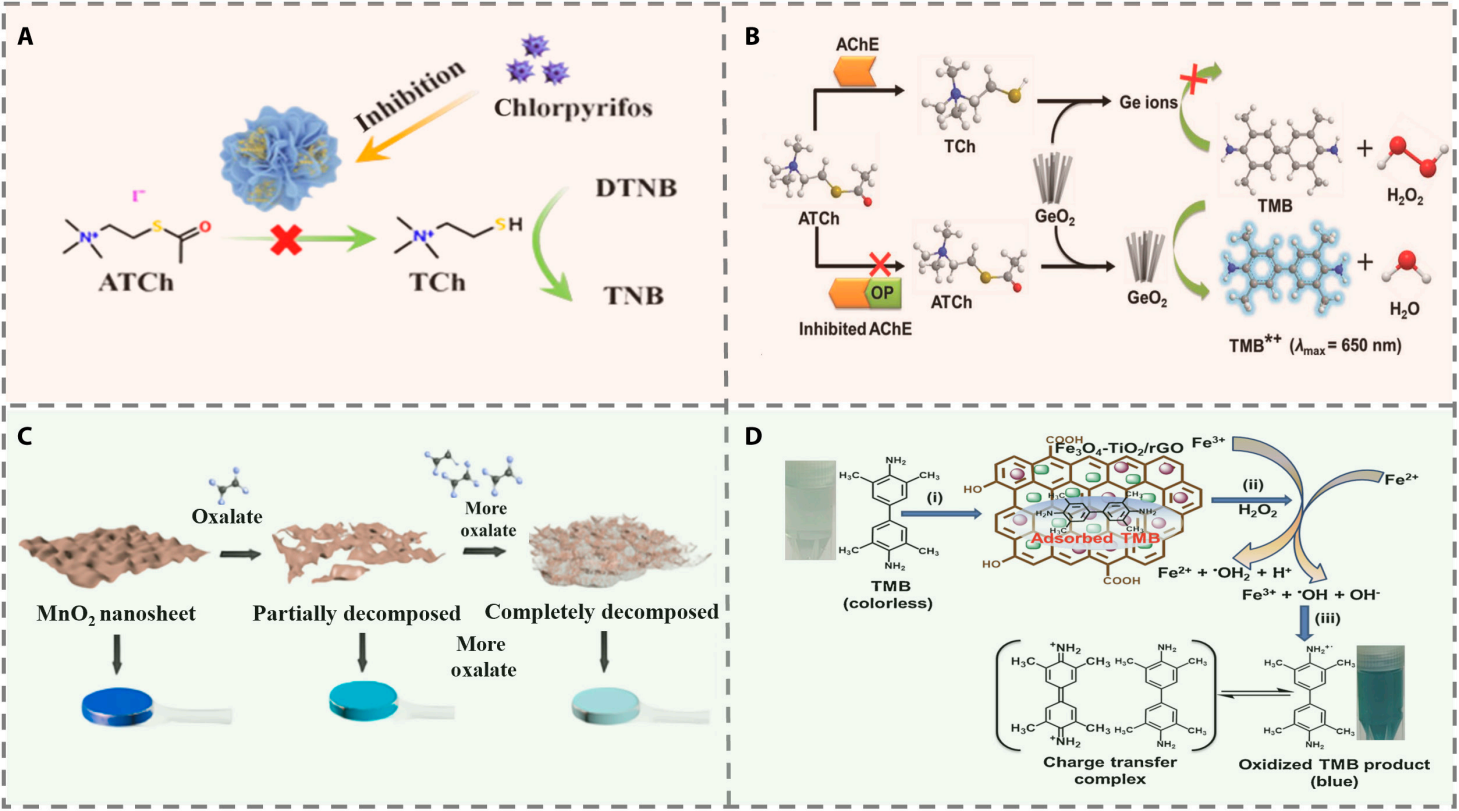
(A) CL sensing platform based on AChE@Zn-MOF-74 [54]. Copyright © 2023 Wiley-VCH GmbH. (B) Schematic illustration of CL sensing strategy [56]. Copyright © 2020 WILEY-VCH Verlag GmbH & Co. KGaA, Weinheim. (C) Schematic illustration of the target-responsive hydrogel platform [57]. Copyright © 2020 Elsevier B.V. All rights reserved. (D) The peroxidase-like activity Fe_3_O_4_-TiO_2_/graphene (FTG) nanocomposite is showing the (i) adsorption of TMB onto FTG nanocomposite surface, (ii) formation of •OH by Fentoneaction, and (iii) TMB oxidation by •OH radicals [60]. Copyright © 2019 Elsevier B.V. All rights reserved.

Enzyme-based CL sensors represent a predominant and efficient strategy for pesticide detection, capitalizing on the selective catalytic activity of enzymes coupled with the tunable optical properties of nanomaterials. Recent advances in this field are largely founded on enzyme inhibition mechanisms, which can be divided into systems employing natural enzymes and artificial nanozymes. In natural enzyme systems, detection often relies on the specific inhibition of AChE by OP pesticides. For example, the in situ encapsulation of AChE within a flower-like Zn-MOF-74 structure significantly enhances enzyme stability through protective confinement and improved mass transport, yielding a 3-fold increase in storage stability at room temperature. By monitoring the absorbance decrease at 412 nm resulting from the reaction between TCh and 2-nitrobenzoic acid (DTNB), this system enables accurate quantification of pesticides such as chlorpyrifos in tomato juice (Fig. 6A). Furthermore, it facilitated the real-time monitoring of pesticide degradation kinetics in this matrix, revealing a half-life of 6.3 h and thereby offering a valuable tool for environmental behavior studies [53].

Nanozyme systems demonstrate remarkable flexibility and innovation. Ultrathin CoOOH nanosheets maintain 95% peroxidase-like activity after 30 d at 4 °C. Coupled with AChE and choline oxidase, they enable inkjet-printed paper sensors for smartphone-based plasma enzyme detection, showing strong correlation (*R*^2^ = 0.991) with Ellman’s method [54]. TCh modulates nanomaterial functionality; bimetallic Mn/Fe-MIL-53 MOFs exhibit oxidase-like activity via Mn^3+^/Mn^4+^ and Fe^2+^/Fe^3+^ redox cycling, oxidizing 3,3',5,5'-tetramethylbenzidine (TMB) to blue oxidized 3,3',5,5'-tetramethylbenzidine (oxTMB). TCh disrupts their structure, an effect reversed by pesticides, allowing methyl parathion detection [55]. Additionally, TCh triggers reductive decomposition of peroxidase-mimetic GeO₂. Pesticides inhibit AChE, leaving residual GeO_2_ to catalyze TMB oxidation, avoiding false positives and achieving femtomolar level detection with superior sensitivity and anti-interference ability (Fig. 6B) [56].

Alternatively, enzyme-free strategies that rely on direct interaction between analytes and nanozyme have been developed. For example, MnO_2_ NS exhibits intrinsic oxidase-mimicking activity, catalyzing the oxidation of colorless TMB to a blue oxidized product. Under acidic conditions, however, oxalate decomposes the MnO_2_ NS, resulting in a reduction of their catalytic activity and a corresponding fade in the blue coloration (Fig. 6C) [57]. Similarly, glutathione–iron (GSH–Fe) nanozymes demonstrate peroxidase-like behavior, facilitating the H_2_O_2_-mediated oxidation of TMB to similarly produce a blue signal. In this case, thiram inhibits nanozyme activity through Fe–S bond-induced surface passivation. Both mechanisms leverage smartphone-based image acquisition, thereby transforming CL data into quantifiable signals and ultimately enabling highly sensitive on-site detection capabilities [58]. Recent research emphasizes multifunctional nanozymes with advanced detection. For instance, Ni–Co nanomaterials with dual enzyme activities detect ziram via direct Ni–S inhibition and AChE suppression, achieving 0.33 μM sensitivity and 96.5% pesticide identification accuracy using smartphone RGB and neural networks [59]. Another system uses Fe_3_O_4_-TiO_2_/rGO to detect atrazine via hydrogen bonding and degrade it under light, maintaining 92% activity after 10 reuse cycles (Fig. 6D) [60].

Enzyme-mediated CL sensing platforms, particularly those incorporating nanomaterials and nanotechnology, effectively merge the specific biorecognition capabilities of enzymes with the enhanced optical properties of nanomaterials. Within the domain of pesticide detection, such integrated systems demonstrate considerable advantages, including exceptional sensitivity, high selectivity, ease of integration into portable formats, and functional versatility. Future advancements in this field are anticipated to focus on intelligent sensing architectures, multifunctional system design, and continued innovation in nanozyme development. However, enzyme-mediated CL sensors also face several challenges, particularly regarding long-term enzyme stability and the accuracy of on-site quantitative measurements. Addressing these limitations requires continuous efforts in advanced materials engineering and fundamental mechanism research.

#### Antibody-assisted methods

Antibody-assisted CL sensing platforms leverage the high specificity of immunorecognition to generate visible color changes that can be readily observed with the naked eye or simple optical devices [61]. These systems preserve the operational simplicity and intuitive visual readout central to CL methods while greatly enhancing selectivity through precise antibody–antigen interactions.

**Fig. 7.**
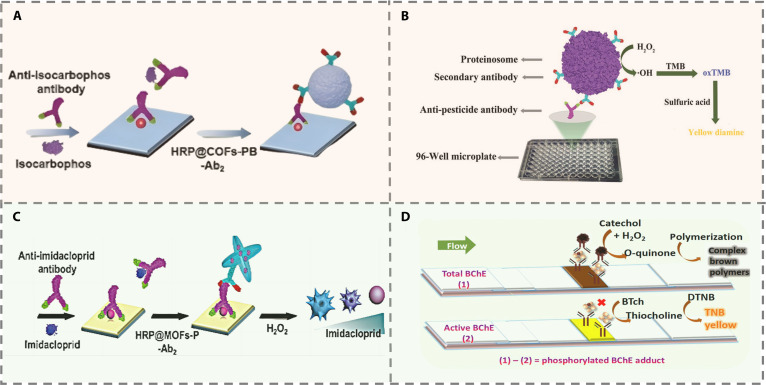
(A) Schematic illustration of HRP@COFs-PB-based HRP@COFs-PB-based immunosensors (CLISA) for imidacloprid detection [62]. Copyright © 2025 Wiley-VCH GmbH. (B) Schematic diagram of function evaluation [63]. Copyright © 2019 WILEY-VCH Verlag GmbH & Co. KGaA, Weinheim. (C) Schematic illustration of HRP@MOFs-P-based immunosensors (HLISA) for imidacloprid detection [64]. Copyright © 2023 Wiley-VCH GmbH. (D) Illustration of the principle of the simultaneous immunodetection of the BChE activity and total amount of BChE [66]. Copyright © 2018 American Chemical Society.

Recent advances in antibody-based optical sensors have led to considerable progress in pesticide detection. HRP-based platforms, in particular, have seen improved performance through material designs and signal amplification strategies. One representative study employed dynamic covalent chemistry to control the spatial distribution of enzymes within covalent COFs, enabling co-encapsulation of HRP and Prussian blue (PB) (Fig. 7A). The resulting composite retained high enzymatic activity even under strongly acidic conditions, demonstrating utility for environmental sample analysis [62]. Our group used a cetyltrimethylammonium bromide (CTAB)-guided self-assembly strategy to form hollow proteinosome at the oil–water interface, into which HRP and secondary antibodies were densely loaded (Fig. 7B). This design significantly increased enzyme loading and led to an ultrasensitive competitive immunoassay for imidacloprid, achieving a detection limit of 1 pg/ml, which is approximately 150 times more sensitive than conventional ELISA [63]. Furthermore, peptide-engineered HRP@MOF nanostructures enhanced substrate diffusion; enzymatic I₂ production etched gold nanostars, inducing a plasmonic shift and color change (Fig. 7C). With smartphone RGB analysis, this method increased imidacloprid sensitivity by 3 orders of magnitude, enabling advanced on-site monitoring [64].

In addition to conventional HRP-based systems, a variety of novel detection strategies have demonstrated notable performance with enhanced capabilities. For example, protein-inorganic hybrid nanoprobes (PIHNs) were fabricated via biomimetic mineralization, embedding antibodies and enzymes within a copper phosphate shell. This configuration preserved 60% of enzymatic activity at 60 °C and enhanced immunoassay sensitivity by 3 orders of magnitude, yielding a detection limit of 0.03 ng/ml while greatly improving reagent stability [65]. A bifunctional test strip integrated with PtPd nanoparticles and butyrylcholinesterase (BChE) antibodies enabled dual-mode detection. PtPd nanoparticles catalyzed catechol polymerization to produce a brown signal indicating total BChE, while the Ellman reaction generated yellow TCh for active BChE quantification (Fig. 7D). Using a smartphone ambient light sensor to measure transmitted light attenuation, this system simultaneously detected total BChE (0.05 to 6.4 nM) and active BChE (0.1 to 6.4 nM), providing comprehensive biomarker profiling for pesticide exposure [66]. Additionally, atomically dispersed dipeptide-stabilized copper nanozymes (His–Cys–Cu) mimicking laccase activity have been developed. Their multivalent copper centers employed a halide ion “recognition antenna” mechanism to enhance substrate specificity. Incorporated into a nanozyme-linked immunosorbent assay (NLISA) and embedded in stimulus-responsive hydrogels, these nanozymes replaced traditional enzyme labels and improved imidacloprid detection sensitivity by 150-fold, highlighting a promising alternative direction for assay development [67].

In summary, antibody-assisted CL platforms achieve ultrasensitive detection (down to pg ml^−1^) via the integration of specific immunorecognition, nanomaterial-enhanced signaling, and innovative amplification strategies, alongside notable stability in challenging environments. These advances significantly improve the sensitivity of pesticide detection and facilitate on-site analysis in complex matrices. However, challenges remain, including the complexity of probe synthesis, the high cost of antibodies and nanomaterials, and the limited stability and reproducibility of integrated systems. Future efforts should focus on simplifying fabrication processes, enhancing system robustness and standardization, and validating performance in real-world samples to bridge the gap between laboratory innovation and practical application.

#### Aptamer-based methods

The integration of aptamer-based recognition with CL sensing platforms preserves the inherent advantages of CL methods, such as intuitive visual readout, minimal instrumental requirements, and ease of operation, while incorporating the unique properties of aptamers, including high specificity and ease of chemical modification. This synergy significantly broadens detection scope and enhances sensor performance, offering promising applications in environmental water pollutant screening, food pesticide residue monitoring, and rapid clinical diagnostics. In recent years, aptamers have driven notable advances in pesticide detection, evolving from foundational research on mechanisms and material design toward sophisticated innovations in signal transduction and integrated device development.

**Fig. 8.**
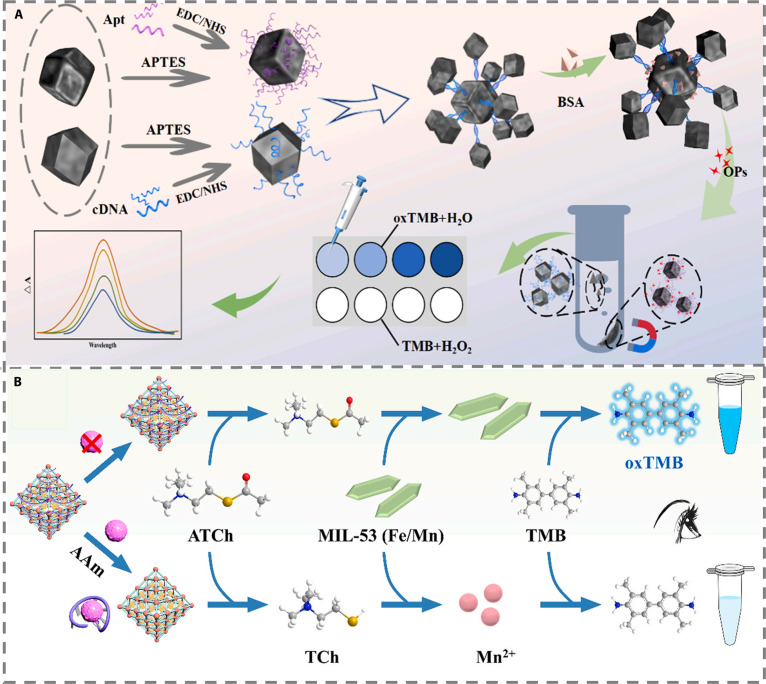
(A) Schematic illustration of the preparation process of Fe–Co magnetic nanoparticles (MNPs) and Fe–N–C nanozymes and the assembly process for the MOF-based aptasensor [68]. Copyright © 2022 Elsevier B.V. All rights reserved. (B) Schematic illustration of the detection principle for acrylamide (AAm) [71]. Copyright © 2024 Elsevier B.V. All rights are reserved, including those for text and data mining, AI training, and similar technologies.

The constraints associated with single-function materials in complex detection scenarios have prompted the development of diverse multifunctional material-based strategies. For instance, a hybrid complex composed of MOF-derived Fe–N–C nanozymes and aptamer-conjugated Fe–Co magnetic nanoparticles capitalizes on the competitive displacement of complementary DNA strand OPs. Coupled with magnetic separation and the catalytic oxidation of TMB and H_2_O_2_, this approach enables highly sensitive detection (Fig. 8A) [68]. Signal transduction mechanisms continue to diversify. A notable example involves cationic AuNPs and an acetamiprid-specific aptamer in a salt-free system: In the absence of the target, aptamer binding induces a blue-shifted aggregation of AuNPs, whereas target presence leads to red-shifted dispersion. Quantification is achieved by measuring the absorbance ratio at 560 and 700 nm [69]. Meanwhile, a high-affinity aptamer developed for quinclorac employs a dye-displacement mechanism for signal amplification. Cyanine 7 dye intercalated within the aptamer yields a light blue solution; upon target-induced G-triplex formation, the released dye forms H-aggregates, causing a rapid color shift to dark blue within 1 min and achieving a detection limit of 237 nM [70].

Advancements in integration have led to increasingly sophisticated systems. An aptamer-mediated nanozyme-amplified lateral flow assay (Apt-CSNLFA) employs Au@Pt core–shell nanoparticles as catalytic probes. Aptamers immobilized at the test line capture these probes to catalyze indophenol blue formation, and incorporation of a sucrose delay mechanism enables one-step automated operation with a detection limit of 0.17 ng/ml, markedly improving efficiency and practicality (Fig. 8B) [71].

Aptamer-nanozyme synergies demonstrate significant potential in pesticide detection, combining precise molecular recognition with strong catalytic activity to achieve ultrasensitive and rapid detection in pg/ml levels. These systems retain CL advantages such as visual readout and smartphone compatibility, while nanozyme stability ensures performance in complex matrices. Functionalization ease facilitates integrated, portable designs. However, challenges such as limited sensitivity in some systems, nonspecific adsorption, sample-specific optimization, high aptamer cost, and inconsistent nanozyme production hinder broad application. Addressing these issues is crucial for practical scalability.

#### Others

In addition to conventional recognition elements, MIPs have emerged as attractive materials for constructing CL sensors, offering high selectivity, chemical stability, ease of operation, and cost efficiency. These attributes make them suitable for diverse applications such as environmental monitoring, food safety, and biomedical diagnostics. Recent advances include an integrated platform combining surface molecularly imprinted polymers (SMIPs), zinc ferrite nanozymes, microfluidic paper-based chips, and smartphone imaging for on-site butachlor detection. This system operates through steric hindrance effects when target molecules bind. However, by combining multiple technologies and selecting key components with the help of quantum chemistry, it becomes highly practical and offers a scalable foundation for future development [72]. Further innovation is exemplified by a bifunctional monomer system employing (3-aminopropyl) triethoxysilane (APTES) and phenyltriethoxysilane (PTES), which enhances binding affinity for 3-phenoxybenzoic acid (3-PBD) through synergistic hydrogen bonding and π–π interactions. This approach introduced an “offline recognition, online detection” strategy, repurposing MIPs as sample preparation and enrichment units rather than direct signal transducers. By coupling MIP-based purification with a potassium permanganate CL system, interference from reducing agents in complex matrices is effectively minimized, thereby expanding the role of supramolecular recognition in practical sensing [73]. A notable shift from material design to functional integration is reflected in core–shell structured sensors such as cobalt–zinc ZIFs, which exhibit enhanced peroxidase-like activity through bimetallic synergy. When coupled with a surface-imprinted layer, dimethoate capture modulates catalytic activity via electron transfer or radical quenching mechanisms rather than steric effects. This atomic-level design enables ultrasensitive detection, over 5 reuse cycles, and demonstrates a favorable balance between performance and cost-effectiveness, signaling a transition from technological hybridation toward materially and functionally integrated systems [74].

CL sensors are simple to operate and low-cost, making them suitable for portable on-site pesticide detection. However, they face key challenges, such as relatively low sensitivity, subjective color-based quantification, and susceptibility to false positives in colored samples. Solving these issues will be a major focus of future research.

### SERS strategies

SERS significantly amplifies Raman signals by adsorbing target molecules onto plasmonic metal substrates like gold or silver. These substrates enhance molecular vibrations, enabling highly sensitive and specific detection. In pesticide analysis, SERS has shown greatly improved sensitivity and is evolving from simple signal enhancement toward intelligent, automated analysis [75]. This progress is driven by 2 key approaches: integrating biorecognition elements and designing multi-dimensional metallic substrates. This section reviews recent SERS sensor advances from these perspectives and their use in environmental, food, clinical, and biomedical application

#### Biorecognition element-based method

**Fig. 9.**
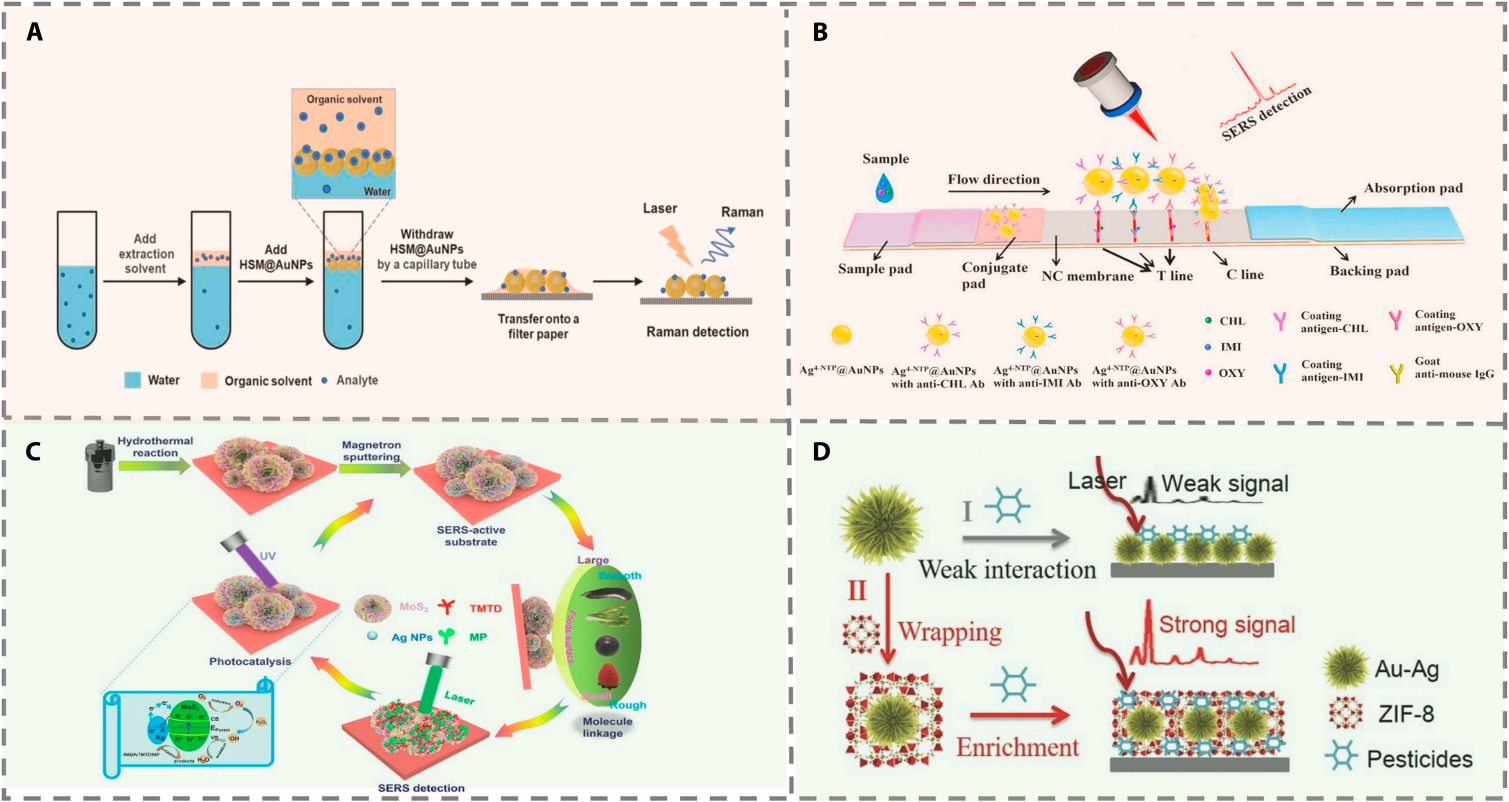
(A) Schematic illustration for the detection of trace pesticides in water based on extraction-integrated hollow silica microspheres (HSM)@AuNPs strategy [76]. Copyright © 2022 Elsevier B.V. All rights reserved. (B) Schematic illustration of the core–shell SERS nanotag-based multiplex LFA strip [77]. Copyright © 2021 Elsevier B.V. All rights reserved. (C) Typical fabrication process of the flower-like MoS_2_@Ag hybrid matrix and their application in recyclable monitoring of pesticide residues [79]. Copyright © 2020 American Chemical Society. (D) Schematic illustration of ZIF-8 encapsulated urchin-like Au–Ag alloyed nanocrystals (UAANs) for trace detection of pesticides [80]. Copyright © 2019 Elsevier B.V. All rights reserved.

The integration of biological recognition elements has endowed SERS with molecular specificity previously unattainable through physical enhancement alone. This integration is exemplified by immunorecognition strategies, where an immunochromatography–SERS platform demonstrates a remarkable 10^3^- to 10^4^-fold enhancement in detection sensitivity compared to conventional ELISAs for pesticide monitoring (Fig. 9A) [76]. Further extending these capabilities, Ag@4-NTP@Au core–shell nanotags coupled with lateral flow immunoassay and an single-stranded DNA (ssDNA)–streptavidin bridging strategy achieve simultaneous quantification of 3 distinct pesticides at sub-nanogram per milliliter concentrations (Fig. 9B) [77].

#### Non-biorecognition element-based methods

In addition to improving sensitivity through integration with biorecognition elements, current research also focuses on enhancing the intrinsic performance of SERS substrates. The research focus has shifted from the mere pursuit of signal amplification to addressing issues of stability, reproducibility, and environmental adaptability in a holistic manner. Gold substrates, valued for their corrosion resistance and biocompatibility, have enabled long-term monitoring in challenging environments. For instance, the development of gold icosahedral nanostars with precisely controlled tip structures demonstrates how morphological tuning can yield enhancement factors exceeding 10^6^, facilitating the establishment of reproducible SERS fingerprint libraries for pesticides [78]. Silver substrates are widely adopted for their strong plasmonic response and cost efficiency. MoS_2_@Ag hybrid materials not only enhance SERS signals by suppressing charge recombination but also introduce photocatalytic degradation functionality, augmenting the sustainability of the method (Fig. 9C) [79]. Bimetallic Au–Ag structures further illustrate the trend toward multifunctional design, integrating the chemical stability of gold with the strong plasmonic response of silver. These are often coupled with porous materials such as MOFs to simultaneously enhance selectivity and sensitivity, as seen in systems achieving parts-per-billion detection of weakly adsorbing pesticides (Fig. 9D) [80].

SERS sensors have ultra-high sensitivity. Combining them with biometric elements improves their specificity. However, key challenges remain. Substrate preparation often lacks repeatability and consistency. Some optimized substrates are costly. The enhancement effect depends strongly on distance, which limits design flexibility. Nonspecific adsorption occurs in real samples. Future research aims to effectively solve these problems.

### Other strategies

Surface plasmon resonance (SPR) and chemiluminescence strategies have gained prominence in pesticide detection due to their efficient operation and high analytical performance. Leveraging specific recognition elements, these methods offer excellent sensitivity and selectivity for real-time monitoring. This chapter subsequently reviews recent representative studies employing SPR and chemiluminescence for pesticide analysis.

#### SPR methods

SPR technology offers significant advantages for detecting environmental pollutants, including label-free operation, real-time monitoring, and high sensitivity. This technique detects molecular binding events by tracking shifts in the resonance condition of surface plasmon waves [81]. Conventional SPR, based on propagating surface plasmons, provides excellent sensitivity but often requires bulky instrumentation. Recent advances focus on platforms such as MIP-SPR and nano-enhanced CL-SPR systems. MIP-SPR sensors utilize molecularly imprinted nanofilms to achieve ultrasensitive detection of pesticides [82]. In contrast, CL-SPR sensors employ metallic nanoparticles to exploit the localized surface plasmon resonance (LSPR) effect, enabling visual detection through target-induced aggregation and color changes [83]. LSPR-based systems benefit from simpler optical configurations, offering a distinct path toward miniaturization and cost-effective on-site analysis.

Compared to conventional methods like LC-MS/MS, SPR provides label-free operation and real-time results without extensive sample preparation. However, challenges remain, including interference from complex matrices, dependence on specific recognition elements, nonspecific adsorption, high costs, and limitations in portability. Future development should prioritize miniaturization and on-site detection capabilities, achieved through the integration of microfluidics, artificial intelligence (AI), and enhanced field applicability.

#### Chemiluminescence methods

Chemiluminescence sensors function through specific chemical reactions wherein analyte recognition triggers a light-emission process, enabling quantification via measurement of the emitted signal intensity. This mechanism affords high sensitivity without requiring external excitation sources, offering benefits such as minimal background interference, rapid response kinetics, operational simplicity, and low instrumental demands. As a result, chemiluminescence has emerged as a key technology for on-site detection of trace-level analytes, particularly pesticides in complex matrices [84].

Recent advances in chemiluminescence sensing have led to diverse strategies based on enzymatic reactions, nanomaterials, plasmonics, and multi-component systems. Enzyme inhibition-based sensors detect OPs by suppressing AChE activity. A foldable paper sensor co-immobilizing AChE, choline oxidase, and HRP shows reduced H_2_O_2_ production and luminol emission, achieving 92% to 99% recovery in vegetables with smartphone compatibility [85]. Similarly, AuNPs/MOFs enhance the luminol-H_2_O_2_ signal via inhibition, reaching 1 nM detection [86]. Nanozymes like ZrO*_x_*-OH mimic phosphatase activity, enabling glyphosate-specific detection with 96.8% to 103.0% recovery [87]. Plasmonic AuNPs allow rapid thiram detection through aggregation-induced quenching (0.35 nM, 40 s) [88]. Multi-component systems such as graphene QDs with KMnO_4_-polyphosphate enable dual-wavelength emission for deltamethrin detection at 0.15 μg/ml, suitable for food safety [89].

Chemiluminescence sensors remain constrained by several limitations. Their performance heavily depends on enzyme activity or nanomaterial specificity, rendering them vulnerable to environmental factors such as enzyme denaturation or nanoparticle aggregation, which compromise signal stability. Complex sample matrices often induce nonspecific luminescence interference through FL quenching or competitive oxidant consumption, adversely affecting specificity. Furthermore, multi-component systems frequently suffer from reproducibility issues and substantial signal variability, impeding reliable quantification.

#### Dual-mode methods

Dual-mode optical sensors combine 2 complementary optical detection techniques to enhance analytical reliability and accuracy. By integrating synergistic signal channels, these systems mitigate limitations inherent in single-mode detection, such as matrix interference, while improving sensitivity and broadening the dynamic range. As a result, they are especially valuable for analyzing complex samples and have garnered significant attention in food safety and environmental monitoring. Common configurations include FL-CL, CL-chemiluminescence, and CL-SERS.

In FL-CL systems, typical strategies utilize MnO_2_-mediated signal modulation and AIE effects. For example, MnO₂–AuNCs–SiO_2_ nanocomposites decompose under ALP, leading to FL recovery and visible solution discoloration. OPs can be detected by inhibiting this process, achieving a detection limit of 0.09 μg/ml [90]. Similarly, AuNCs@ZIF-8 platforms exploit the confinement effect of the ZIF to enhance FL. In the presence of hydrogen peroxide, the FL quenching and CL oxidation of TMB occur concurrently, but both signals are reversed by pesticide inhibition [91]. Nanozyme systems, such as platinum–palladium nanoparticles decorated graphitic carbon nitride nanosheets (PtPd NPs@g-C_3_N_4_), further enable simultaneous modulation of FL and chemiluminescence, allowing highly sensitive detection of organophosphorus compounds [92]. CL-chemiluminescence dual-mode systems often leverage the multi-catalytic activity of nanozymes. For instance, Fe₃O₄@Cu bifunctional nanozymes catalyze both the oxidation of TMB, which yields a color shift from colorless to blue, and the luminol chemiluminescence reaction. Glyphosate chelates Fe/Cu active sites, consuming hydroxyl radicals and inhibiting both CL and luminescence responses, with a detection limit of 0.019 μg/ml [93]. Immunochromatographic test strips that incorporate advanced materials such as g-C_3_N_4_/BiFeO_3_ [94] or MnO_2_ nanoflowers [95] provide dual readout signals, including visible CL bands for qualitative assessment and chemiluminescence for quantitative analysis, with detection limits as low as 33 pg/ml in environmental and food samples. CL-SERS configurations employ hybrid materials such as MIPs with AuNPs (MIPs-AuNPs) to achieve high-selectivity dual-mode detection. These systems permit preliminary visual screening via color change, followed by confirmatory SERS-based quantification of trace analytes. In complex matrices such as fruit juices, detection limits of 1.2 μg/l can be attained within 25 min [96].

These dual-mode strategies substantially enhance detection reliability, sensitivity, and applicability through complementary optical signals, proving particularly effective for rapid trace pollutant analysis. Nevertheless, challenges remain: the inherent complexity and instability of multi-probe designs, susceptibility to matrix interference causing asynchronous signal responses, and a reliance on specific enzymes or nanomaterials that are sensitive to environmental conditions. Moreover, the intricate fabrication and higher operational complexity limit large-scale practical deployment.

## Conclusions and Perspectives

This review systematically examines recent advances in optical sensing technologies for pesticide detection. These sensing platforms have achieved highly sensitive identification of trace pesticide residues through synergistic strategies such as enzyme-catalyzed amplification cascades, plasmonic nanomaterials, confinement-effect nanomaterials, and dual-mode detection architectures. The incorporation of engineered biological receptors and biomimetic materials has substantially improved molecular discrimination among structurally analogous compounds, thereby enhancing detection specificity. Furthermore, the integration of smartphone-based algorithms, flexible substrates, and portable sensing devices has significantly reduced dependence on conventional laboratory infrastructure, overcoming key barriers to real-time field detection and facilitating the implementation of dynamic monitoring paradigms. At the technical level, each optical sensing method presents distinct trade-offs (Table 2). FL sensors offer high sensitivity, but their long-term utility is constrained by photobleaching. In complex samples, issues such as autofluorescence and quenching can further compromise reliability. Additionally, these sensors often require sophisticated instrumentation, which increases operational costs, while spectral overlap limits their effectiveness in multiplex detection. In contrast, CL sensors are well-suited for rapid on-site screening, although they generally lack sufficient quantitative accuracy. Their sensitivity tends to be lower than that of FL sensors or SERS. Moreover, false positives are common when testing naturally colored samples. SERS provides a unique molecular fingerprinting capability. However, its application is often hampered by the reproducibility and uniformity of SERS substrate fabrication, the high cost of some optimized substrates, the distance-dependent nature of the enhancement, which can limit design flexibility, and issues with nonspecific adsorption in real samples. SPR allows for label-free, real-time monitoring but is vulnerable to nonspecific adsorption. Finally, dual-mode sensing improves reliability through signal cross-validation, albeit at the cost of increased system and probe design complexity. Understanding these characteristics is crucial for selecting the appropriate technology for specific pesticide detection scenarios.

**Table 2.** Comparison of the analytical performance of various optical biosensors

Optical detection technology	Limit of detection (LOD)	Response time	Actual sample suitability	Reference
Fluorescence (FL)	10^−9^–10^−12^ M	Millisecond to minute scale	Suitable for detecting biomolecules, heavy metal ions, and environmental pollutants; enables multi-channel multiplexing, and is often used in clinical diagnosis and environmental trace analysis.	[15–45]
Colorimetry (CL)	10^−6^–10^−9^ M	Minute scale	Suitable for on-site rapid detection; features simple operation and no need for complex instruments, and is commonly used in qualitative/semiquantitative analysis of food additives, pesticide residues, etc.	[54–72]
Surface-enhanced Raman spectroscopy (SERS)	10^−12^–10^−15^ M	Second to minute scale	Applicable for molecular structure identification and trace detection; can distinguish isomers, and is widely used in pharmaceutical analysis, food safety, cultural relic identification, etc.	[77–81]
Surface plasmon resonance (SPR)	10^−7^–10^−10^ M	Millisecond to second scale	Enables label-free, real-time detection of biomolecular interactions (e.g., antigen–antibody binding, protein–ligand interactions); widely used in drug discovery, biosensing, and clinical immunoassays.	[83,84]
Chemiluminescence	10^−9^–10^−12^ M	Second to minute scale	Simple structure; low cost; suitable for development into portable devices, on-site rapid detection equipment, or test strips	[86–90]
Dual-mode detection	10^−10^–10^−14^ M	Second to minute scale	Suitable for complex sample matrices (e.g., blood, environmental water), and applied in ultra-trace pollutant monitoring, precision medicine, and multi-analyte simultaneous detection	[91–96]

Future advancements should focus on several key priorities. In fundamental research, it is essential to develop robust biorecognition elements, such as synthetic enzyme analogs, single-atom catalysts, and covalently stabilized aptamer-based frameworks that enhance detection sensitivity. At the device level, the use of degradable nanomaterials and flexible electronics could enable wearable patches and disposable sensors suitable for field applications. Technologically, integrating machine learning with microfluidic systems will facilitate in situ sample purification and high-throughput multiplexed detection. AI is set to play a transformative role by improving spectral analysis, enabling predictive modeling, and supporting autonomous decision-making. When embedded in sensing platforms and connected via Internet of Things (IoT) networks, these advances can form intelligent systems for real-time, on-site pesticide monitoring. There is also a critical need to develop green sensors through sustainable designs that incorporate biodegradable and eco-friendly components. This approach helps reduce environmental impact after disposal and addresses growing sustainability concerns. Furthermore, establishing standardized evaluation databases and validated on-site testing protocols will be vital for regulatory approval and technological interoperability. Ultimately, interdisciplinary collaboration across materials science, micro/nanofabrication, and AI is expected to create intelligent monitoring networks that span the entire agricultural supply chain, thereby supporting global food security and ecological sustainability.
